# Preferential Colonization of Osteoblasts Over Co-cultured Bacteria on a Bifunctional Biomaterial Surface

**DOI:** 10.3389/fmicb.2018.02219

**Published:** 2018-10-02

**Authors:** Linyang Chu, Ying Yang, Shengbing Yang, Qiming Fan, Zhifeng Yu, Xi-Le Hu, Tony D. James, Xiao-Peng He, Tingting Tang

**Affiliations:** ^1^Shanghai Key Laboratory of Orthopedic Implants, Department of Orthopedic Surgery, Shanghai Ninth People’s Hospital, Shanghai Jiao Tong University School of Medicine, Shanghai, China; ^2^Department of Plastic Surgery, Xiangya Hospital, Central South University, Changsha, China; ^3^Key Laboratory for Advanced Materials and Feringa Nobel Prize Scientist Joint Research Center, East China University of Science and Technology, Shanghai, China; ^4^Department of Chemistry, University of Bath, Bath, United Kingdom; ^5^Department of Materials and Life Sciences, Faculty of Science and Technology, Sophia University, Tokyo, Japan

**Keywords:** co-cultured system, competitive colonization, bifunctional surface, *Staphylococcus aureus*, osteoblast

## Abstract

Implant-related infection is a devastating complication in clinical trauma and orthopedics. The aim of this study is to use a bifunctional biomaterial surface in order to investigate the competitive colonization between osteoblasts and bacteria, which is the cause of implant-related infection. A bone-engineering material capable of simultaneously facilitating osteoblast adhesion and inhibiting the growth of *Staphylococcus aureus* (*S. aureus*) was prepared. Then, three different co-cultured systems were developed in order to investigate the competitive colonization between the two cohorts on the surface. The results suggested that while the pre-culturing of either cohort compromised the subsequent adhesion of the other according to the ‘race for the surface’ theory, the synergistic effect of preferential cell adhesion and antibacterial activity of the bifunctional surface led to the predominant colonization and survival of osteoblasts, effectively inhibiting the bacterial adhesion and biofilm formation of *S. aureus* in the co-culture systems with both cohorts. This research offers new insight into the investigation of competitive surface-colonization between osteoblasts and bacteria for implant-related infection.

## Introduction

The quest to discover bone graft materials with excellent osteogenesis and antibacterial activity has been a global challenge (Arciola et al., 2012; Kumar et al., 2016; Ouyang et al., 2016). Despite the numerous surgical approaches developed for the implementation of bone void fillers and bone-related biomaterials (Sheikh et al., 2015; Tang and Qin, 2016), the remaining technical and surgical shortcomings are substantial. In particular, bacterial contamination is inevitable for almost all bone-graft materials and medical devices (Arciola et al., 2011; Tande and Patel, 2014). To date, the fight against implant-related infections is far from satisfactory. After the implantation of an endo-prosthesis, pathogens can initiate adhesion onto the implant surface forming bacterial biofilms, which are responsible for the protection of pathogens from host immune defense and antibiotic treatment (Grassi et al., 2017; Abinandan et al., 2018; Del Pozo, 2018). As a result, advanced bioactive materials that can facilitate cell adhesion while inhibiting bacterial colonization are of great clinical significance.

According to the ‘race for the surface’ theory, the presence of a foreign body triggers a race between bacteria and host cells for colonization on the surface of the implant (Gristina, 1987; Gristina et al., 1988; Busscher et al., 2012). Briefly, if the race is won by host cells, the surface is covered by the cells and is less vulnerable to bacterial colonization. On the other hand, if the race is won by bacteria, the implant surface will eventually be covered by biofilms, and the host cells are hampered by bacterial virulence factors and toxins, ultimately leading to infection. The outcome of this race largely determines the severity of infection and tissue integration. Given the resistance of biofilms against host defenses and conventional antibacterial agents, the majority of implant-associated infections are chronic and responsible for implant failure. The colonization of either bacteria or cells on biomaterials has often been addressed as separate issues (Dexter et al., 2001). However, the influence of bacterial contamination on the subsequent cell fate including adhesion, spread and growth on the biomaterials surface in a system where both cohorts exist (which is more relevant to the real-world *in vivo* condition for implants), has hardly been investigated.

**SCHEME 1:**
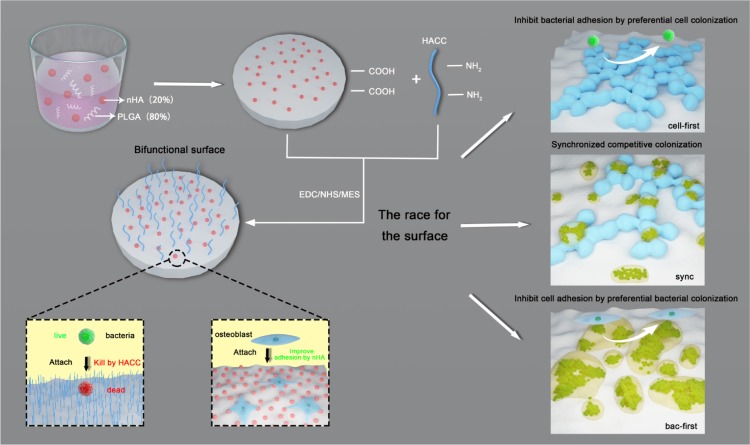
Illustration of the bifunctional PLGA/nHA/HACC composite and the three different co-cultured systems established in order to investigate the competitive surface-colonization.

In our previous study (Yang et al., 2016b), we developed a bone engineering material composed of polylactide-*co*-glycolide (PLGA), hydroxyapatite (HA) and quaternized chitosan (HACC). We determined that HA could promote osteoblasts adhesion and colonization, and HACC (Tan et al., 2012a; Yang et al., 2017), a water-soluble chitosan derivative, exhibited excellent antibacterial activity. Therefore, the PLGA/HA/HACC surface is bifunctional since it improves osteoblasts integration and inhibits bacterial invasion simultaneously (Yang et al., 2016b). Here, using the bifunctional surface, we further developed three different co-cultured systems (i.e., cells-first, bacteria-first, and simultaneous presentation of both cells and bacteria) to investigate the competitive colonization of the two cohorts for implant-related infections (**Scheme 1**). We hypothesized that the bifunctional implant surface could promote the cell to win the race in the co-culture systems with both cohorts which is close to the *in vivo* situation.

## Materials and Methods

### Materials

PLGA (LA/GA: 75/25, average molecular weight: 20 × 10^4^ Da, Shandong Medical Instruments Institute, China) and nHA (nano-hydroxyapatite, particle size < 200 nm, Sigma, United States) were co-dissolved in 1,4-dioxane; the mass ratio of PLGA/nHA was 8/2. HA nanoparticles were used because their nanoscale size may lead to a more uniform dispersion of HA in PLGA. Then the solution was vigorously stirred and sonicated in an ultrasonic bath at 150 W (B3500S-MT, China) with a frequency of 50 Hz for 2 h to disperse nHA homogeneously in PLGA. The prepared pastes were placed in vacuum drying chamber for 12 h to remove 1,4-dioxane, producing a PLGA/nHA solid, which was cut into rounded membranes with a diameter of 10 mm. HACC with a 26% DS of quaternary ammonium was prepared by combining chitosan and glycidyl trimethylammonium chloride (GTMAC, Sigma), as previously reported (Tan et al., 2012b). Following the fabrication of PLGA/nHA membrane, HACC with a mass concentration of 0.2 wt% was dissolved in 50 mL methyl ester sulfonate buffer (MES, Sigma) with 0.04 g 1-(3-dimethylaminopropyl)-3-ethylcarbodiimide hydrochloride (EDC, Sigma) and 0.097 g *N*-Hydroxyl succinimide (NHS, Sigma). Then the mixture and PLGA/nHA membrane was allowed to undergo covalent grafting at 40°C for 12 h. The HACC-grafted membrane (PLGA/nHA/HACC) was sonicated for 5 min and washed with deionized water to remove the excess of linking reagents and un-grafted HACC. Control PLGA and PLGA/nHA membranes without HACC were also manufactured in a similar manner. All three membranes were 1 mm thick and were dried by exposure to an ethanol gradient, coated by gold sputtering, then examined using a scanning electron microscope (SEM, HITACHI SU8220, Japan) at an electron acceleration voltage of 1.5 kV in secondary electron detection mode. The element distribution was determined using energy dispersive spectroscopy (EDS) at an electron acceleration voltage of 1.5 kV. All prepared samples were sterilized by 25 kGy irradiation before use.

### Cell, Bacteria and Modified Culture Medium

The MC3T3-E1 cell-line (a mouse pre-osteoblast line derived from mouse calvaria) was used since the cells are resilient and can rapidly proliferate (Ma et al., 2014). The cells were cultured in cell medium (90% DMEM with high glucose + 10% FBS, PA, United States), maintained at 37°C in a humidified 5% CO_2_ atmosphere and passaged at 70–90% confluency using trypsin–EDTA (PAA, Germany). *Staphylococcus aureus* (ATCC 25923), purchased from the American Type Culture Collection (Manassas, VA, United States), is a biofilm-producing bacteria strain as verified by previous studies (Yang et al., 2016b). The strain used in this study was streaked on an agar plate and grown overnight at 37°C. The plate was then kept at 4°C. A colony was inoculated in 10 mL of tryptone soy broth (TSB) and cultured for 24 h. Subsequently, bacteria were harvested by centrifugation at 5000 *g* for 5 min and sonicated in PBS for 10 s in order to break bacterial aggregates. The suspension was further diluted to the required concentrations for the experiments.

For the co-cultured system, a modified culture medium was developed. Briefly, MC3T3-E1 cells and *S. aureus* were cultured separately in well plates by varying ratios of modified culture medium. Eleven different mixed media were used with standard cell medium percentages of 0, 10, 20, 30, 40, 50, 60, 70, 80, 90, 100% and TSB constituting the remaining fraction. MC3T3-E1 cells and *S. aureus* were inoculated into each mixed medium with a starting concentration of 10^5^ cells/mL and 10^5^ CFUs/mL, respectively. The osteoblasts were resuspended and quantified using a hemocytometer under a light optical microscope (Olympus, Germany) after incubation for 24 h. The number of *S. aureus* CFUs was counted by spread plate method after 24 h. The mixed medium that best promoted growth of both osteoblasts and *S. aureus* was denoted as ‘modified culture medium’ and was subsequently used as the co-cultured system.

### Co-cultured Systems

PLGA, PLGA/nHA and PLGA/nHA/HACC membranes were placed in 12-well plates with modified culture medium. Three different co-cultured systems were prepared as follows: (1) cells preferentially inoculated before bacteria (cell-first group), (2) bacteria preferentially inoculated before cells (bac-first group) and (3) both simultaneously inoculated (sync group). The preferentially inoculated cells or bacteria were left to stand for 2 h to complete the initial steady adhesion and then the other was added subsequently in order to simulate the possible situations in the race of competitive colonization. The mixture was incubated for 24 h after the cells and bacteria were co-presented in the co-cultured system. At the end of co-culture, the samples were washed twice with PBS to remove planktonic bacteria and cells.

In the preparation stage for the co-cultured system, four bacterial concentrations (10^6^, 10^5^, 10^4^, and 10^3^ CFUs/well) and five cell concentrations (10^4^, 5 × 10^4^, 10^5^, 2 × 10^5^, 5 × 10^5^ cells/well) were used to determine the optimal ratio of bacteria to cells. Finally, a ratio of 1:20 (10^4^ CFUs/well, 2 × 10^5^ cells/well) was used for all co-cultured experiments because higher bacterial concentrations or an improper ratio would lead to a rapid decline of cells rendering dynamic observations impossible (**Table 1**).

**Table 1:** Serial bacterial and cell concentrations to determine the optimal ratio of bacteria to cells in the co-cultured systems.


Cells/well	10^4^	5 × 10^4^	10^5^	2 × 10^5^	5 × 10^5^
CFUs/well
10^3^	^∗^	>5 × 10^3^	>10^4^	>6 × 10^4^	>1.5 × 10^5^
10^4^	^∗^	<5 × 10^3^	<10^4^	>3 × 10^4^	>8 × 10^4^
10^5^	^∗^	^∗^	^∗^	^∗^	^∗^
10^6^	^∗^	^∗^	^∗^	^∗^	^∗^


*The results in table represented the live cells after co-culturing, ^∗^no cells were detected.*

### Competitive Colonization Assay in Co-cultured Systems

After co-culturing, SEM was used for evaluating the topography of osteoblasts and bacteria on the three samples. At each time point, the samples were fixed in 2.5% glutaraldehyde solution, washed three times with PBS, and dried through an ethanol series. Then the samples were coated by gold sputtering and examined by SEM. Moreover, the samples were stained for 2 min with Acridine Orange (BD, United States), and rinsed with PBS to remove excessive dye molecules. The samples were observed by confocal laser scanning microscopy (CLSM, Leica TCS SP8, Leica Microsystems, Germany).

To further study the competitive colonization in co-cultured system, the spreading, adhesion and focal adhesions formation of osteoblasts on biomaterial surface were analyzed. Briefly, the samples were fixed in 4% paraformaldehyde for 15 min, permeabilized with 0.1% Triton X-100 in PBS for 10 min and blocked with 5% albumin from bovine serum albumin (BSA). Then rabbit monoclonal antibody to vinculin (ab196454, Abcam) was used to stain the focal adhesions. Meanwhile, 4,6-diamidino-2-phenylindole (DAPI, Sigma) was used to stain the cell nuclei and rhodamine phalloidin (Molecular Probe, Cytoskeleton) was used to stain the filamentous actin of the cytoskeleton for analysis of cell spreading and adhesion. The samples were observed by CLSM.

### Live/Dead Cells Assay and Quantitative Analysis

The cell viability of the various sample surfaces was analyzed by a Live/Dead Cell kit (ab115347, Abcam, United Kingdom) as described previously (Yang et al., 2016a). After co-culturing for 24 h, the samples were stained with 500 μL of a combination dye for 10 min, and then detected by CLSM. The viable cells with esterase activity appeared green, whereas dead cells with compromised plasma membranes appeared red, as described in the manufacturer’s protocol. Moreover, the osteoblasts adhering to the sample surfaces were removed using trypsin-EDTA from the co-cultured system. The solution was centrifuged at 900 rpm for 4 min and the deposited cells were washed with PBS. A cell counting kit-8 (CCK-8, Dojindo, CK04-500, Japan) was used in this experiment to quantitatively evaluate the cell viability after co-culturing. Then 100 μL of cells resuspension solution and 10 μL of CCK-8 solution were added to 96-well plates followed by incubation at 37°C for 2.5 h. The optical density (OD) at 450 nm was determined using a microplate reader (BIOTEK, United States) as published previously (Yang et al., 2016b).

### Live/Dead Bacteria Assay and Quantitative Analysis

The bacterial viability on various sample surfaces was analyzed using a Live/Dead BacLight viability kit (Thermo Fisher Scientific, United States) (Yang et al., 2016a). Briefly, after co-culturing for 24 h, the samples were stained with 500 μL of a combination dye and visualized by CLSM. Live bacteria with intact cell membranes displayed green fluorescence, whereas dead bacteria with damaged cell membranes displayed red fluorescence. Meanwhile, the various samples were placed in glass test tubes containing 1 mL PBS and then sonicated in an ultrasonic bath for 5 min, followed by rapid vortex mixing (Vortex Genie 2, United States) at maximum power for 1 min to thoroughly dislodge the adhered bacteria. The bacteria collected was quantified using serially diluted 10-fold solutions used for determination of CFUs by the spread plate method (Yang et al., 2016b).

### Cell Cytotoxicity Induced by Bacteria

The cells in the co-cultured system were labeled with Alexa Fluor 488 Annexin V and propidium iodide (PI) using the Dead Cell Apoptosis Kit (V12241, Thermo Fisher Scientific, United States) for flow cytometry according to the manufacturer’s instructions (Yang et al., 2016b). The cell collection procedures were like those of the cell quantitative analysis. Based on light scattering, samples were run on a BD LSRFortessa (BD Biosciences) and data were analyzed using the FlowJo software (Tree Star, United States).

In addition, cytotoxicity was determined by assessing the release of cytosolic enzyme lactate dehydrogenase (LDH) into the supernatant. The LDH assay was performed using LDH Cytotoxicity Assay Kit (Beyotime, China) following the manufacturer’s instructions. Supernatants (experimental LDH release, E), samples treated with the lysis solution (maximal LDH release, M) and control samples (spontaneous LDH release, S) were transferred to 96-well plates. The absorbance at 490 nm was read using a microplate reader. The percentage of cytotoxicity values were calculated by the following equation:

Cytotoxicity (%)=100×(E−S)/M

### Analysis of Bacterial Biofilm Formation

Biofilm-related genes (Shanks et al., 2005; Peng et al., 2011; Tan et al., 2012b, 2015) were quantitatively analyzed by the Real-time PCR for bacterial biofilm formation analysis in co-cultured system. Briefly, the bacteria adhering on the samples surfaces were harvested in a similar way to those of the bacterial quantitative analysis after co-culturing for 24 h. Then the bacteria were scraped into the RNA protect bacterial reagent (Qiagen, Germantown, MD, United States) to ensure RNA integrity. Bacteria were pelleted by centrifugation and resuspended in 200 μL TE buffer (10 mM Tris-HCl, 1 mM EDTA, pH 7.0) containing 100 μg/ml lysostaphin (Sigma) and incubated at 37°C for 10 min. Total RNA was isolated using an RNeasy Mini Kit (Qiagen) according to the manufacturer’s instructions, and then DNase I (Invitrogen) was used to eliminate residual genomic DNA. One microgram of total RNA was reversely transcribed using a First Strand cDNA Synthesis Kit (MBI). Real-time PCR was performed on an ABI 7500 Fast machine (Applied Biosystems, France). The reactions were performed using cDNA templates and primers synthesized commercially (**Table 2**). The expression levels of *icaA*, *icaD*, *hld* and *spa* were evaluated and normalized to the internal standard gene *16S rRNA*. The quantification of gene expression was based on the CT value, which was calculated as the average of three replicates for each sample.

**Table 2:** Primer sequences used for real-time PCR.

Target gene	Primers (5′-3′)	Length
*icaA*	F: AACAAGTTGAAGGCATCTCC	20
	R: GATGCTTGTTTGATTCCCT	19
*icaD*	F: ATCGTTGTGATGATTGTTTA	20
	R: GATATGTCACGACCTTTCTT	20
*hld*	F: AAGAATTTTTATCTTAATTAAGGAAGGAGTG	31
	R: TTAGTGAATTTGTTCACTGTGTCGA	25
*spa*	F: GCAAACGGCACTACTGCTGA	20
	R: CACCAGTTTCTGGTAATGCTTGAG	24
*16S rRNA*	F: TCGTGTCGTGAGATGTTGGGTTA	23
	R: GGTTTCGCTGCCCTTTGTATTGT	23


*F, forward. R, reverse.*

### Statistical Analysis

All data are expressed as Mean ± SD. Non-parametric test (Mann–Whitney *U* test), one-way analysis of variance (ANOVA) and the least significant difference (LSD) test were utilized to determine the level of significance; *p* < 0.05 was defined as statistically significant, and *p* < 0.01 was considered highly statistically significant. All statistical analyses of the data were performed using SPSS software (v19.0, United States).

## Results and Discussion

### Development of the Co-cultured Systems

**FIGURE 1:**
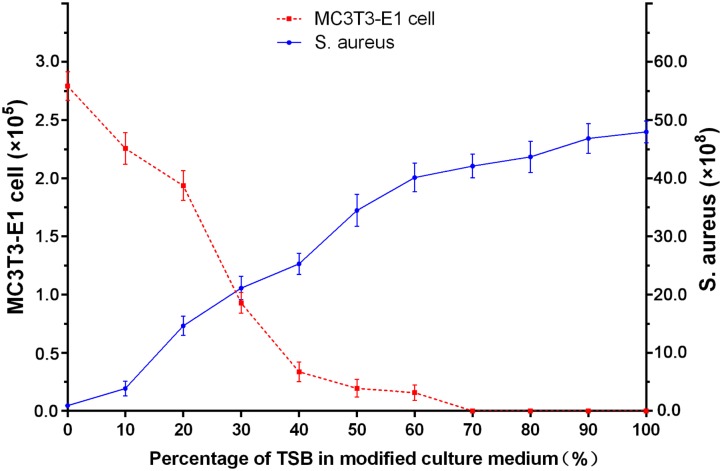
Growth of MC3T3-E1 cells and *S. aureus* in varying ratios of standard cell medium to TSB after 24 h incubation.

In this study, we established three different co-cultured systems in order to investigate the competitive colonization between osteoblasts and *S. aureus* on a bifunctional surface (**Scheme 1**). Although the MC3T3-E1 cells are not entirely clinically relevant, we chose these cells because they have a more stable cell phenotype and less individual variation, thus possessing higher repeatability for experiments. Unlike previous studies (Lee et al., 2010; Foss et al., 2015; McConda et al., 2016), our systems include a bac-first group (material surface was infected before cell adhesion), a cell-first group (cell adhesion before infection), and a sync group (both cohorts are present simultaneously for competition). These systems may provide novel insights into implant-related infections since all three cases could be possible *in vivo*. Furthermore, a new modified culture medium was developed for the co-cultured system. Shown in **Figure 1** are the different culture media (mixture of different ratios of standard medium and TSB) used for culturing the MC3T3-E1 cells and *S. aureus*. While no obvious change in the cell count for MC3T3-E1 was observed in media containing 0–20% TSB, a sharp decreased in cell numbers was detected when the TSB ratio exceeded 30%. Meanwhile, the growth of *S. aureus* was unfavorable when the TSB content was less than 20%. According to the above results and the fact that bacteria proliferate better than cells, a culture medium consisting 80% standard cell medium and 20% TSB was selected for the co-cultured systems. In this medium, the growth of both MC3T3-E1 cells and *S. aureus* was relatively stable.

### Competitive Colonization in Co-cultured Systems

**FIGURE 2:**
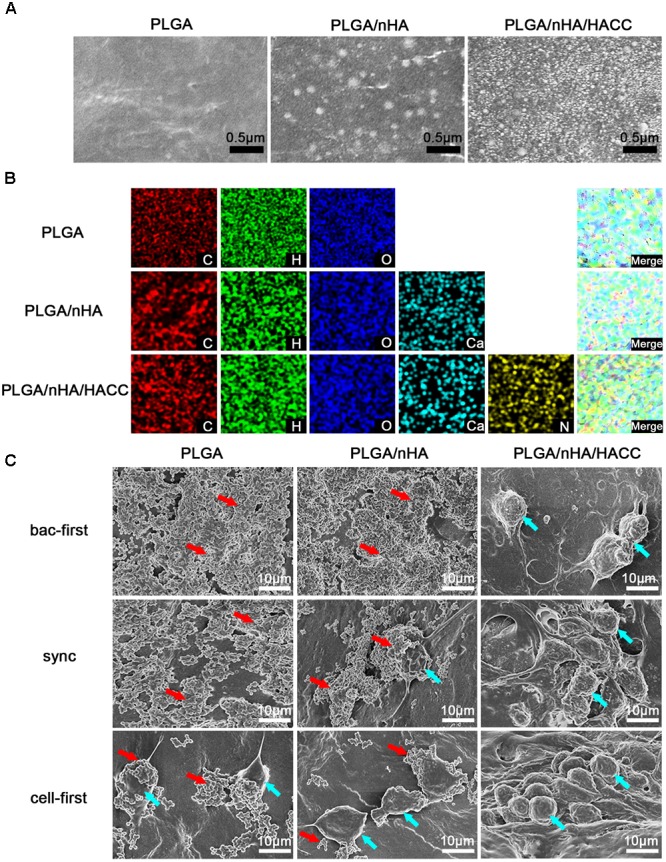
**(A)** SEM images of the different materials surfaces. **(B)** EDS mapping showing the C, H, O, Ca, and N elements of different materials. **(C)** SEM observations of MC3T3-E1 cells and *S. aureus* morphology on different materials surfaces in different co-cultured systems. Blue arrows indicate cell colonization and red arrows indicate bacterial colonization in the image.

The concept of ‘the race for the surface’ has been embraced by many researchers (Subbiahdoss et al., 2009; Pham et al., 2016; Perez-Tanoira et al., 2017), but there has hardly been any experimental methodology established to mimic the plausible situations of competitive colonization. Successful colonization of osteoblasts on implant surface may induce osteogenesis while bacterial colonization could lead to infection (Subbiahdoss et al., 2010; Martinez-Perez et al., 2017). Furthermore, the race of competitive colonization is influenced by the properties of the biomaterial surface (Zaatreh et al., 2016). Several surface-modification methods have been developed to prevent bacterial adhesion and biofilm formation in culturing assays (Rochford et al., 2014; Hindie et al., 2017). In this study the bifunctional PLGA/nHA/HACC biomaterial, where nHA facilitates cell adhesion (Yang et al., 2016b) and HACC has antibacterial activity, was used to test the competitive colonization between cells and bacteria. The surface morphology and elemental distributions of the different materials are shown in **Figures 2A,B**. The surface modified by PLGA/nHA and PLGA/nHA/HACC was observed to be rougher than that by PLGA, which might be due to the presence of nHA and the surface-grafted HACC. The EDS results showed that the Ca elements derived from nHA and the N elements derived from HACC were detected in the PLGA/nHA and PLGA/nHA/HACC groups, which suggests the successful formation of the composites.

Scanning electron microscope (**Figure 2C**) was also used to qualify the difference in surface morphology, composition, and topography of the composites with MC3T3-E1 cells and *S. aureus*. In PLGA (bac-first) and PLGA/nHA (bac-first) groups, there was a large number of colonized *S. aureus* and no cell growth was detected (red arrow, **Figure 2C**). However, in PLGA (cell-first) and PLGA/nHA (sync, cell-first) groups, cell colonization was extensively observed on the surface (blue arrow, **Figure 2C**), and the cells displayed a polygonal morphological change that tend to spread well on the surface. Meanwhile, a small number of *S. aureus* was also observed for both groups. These results are consistent with ‘the race for the surface’ hypothesis (Gristina, 1987; Gristina et al., 1988; Busscher et al., 2012) that when bacteria are preferentially adhered onto the surface, a mass proliferation would subsequently occur, thereby inhibiting cell adhesion. In contrast, if cells are preferentially adhered, the surface is less vulnerable to bacterial colonization. More interestingly, in all the PLGA/nHA/HACC groups, no *S. aureus* was found on the material surface, suggesting that the antibacterial activity of HACC could effectively suppress bacterial colonization in the co-cultured system.

**FIGURE 3:**
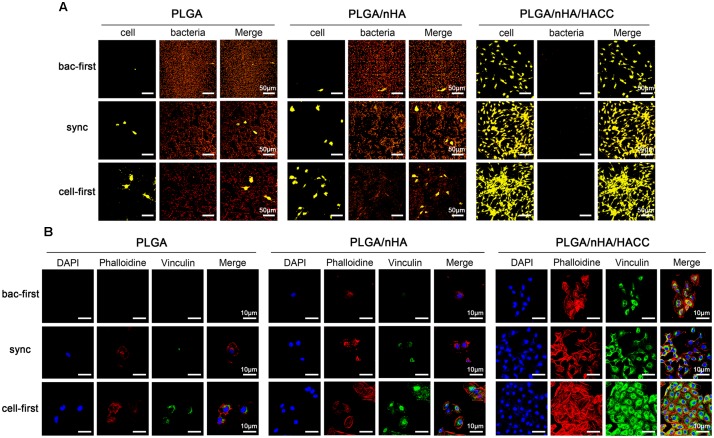
Competitive colonization assay on different materials surfaces in different co-cultured systems. **(A)** Acridine orange staining of cells and bacteria on the surfaces. Representative images of cells (greenish-orange fluorescence) and bacteria (reddish-orange fluorescence). **(B)** The cytoskeletal morphology and focal adhesion formation of cells on surfaces. Representative images of cells stained with DAPI for cell nuclei (blue fluorescence), rhodamine phalloidin for actin filaments (red fluorescence), and monoclonal antibody to vinculin for staining the focal adhesions (green fluorescence).

The competitive colonization between cells and bacteria in the co-cultured systems was also tested by acridine orange staining (**Figure 3A**). While in all PLGA and PLGA/nHA groups, we observed co-existence of bacteria (reddish-orange, **Figure 3A**) and cells (greenish-orange, **Figure 3A**) on the surfaces, in PLGA (cell-first) and PLGA/nHA (cell-first) groups, more osteoblasts and less bacteria colonization were found due to preferential cell adhesion. In all PLGA/nHA/HACC groups, no bacterial colonization on the surfaces was observed. Comparing to the sync and cell-first groups, the PLGA/nHA/HACC (bac-first) group showed relatively less cell colonization on the surface. This could be ascribed to the preferential inoculated bacteria in the surrounding culturing circumstance, which interrupted the subsequent cell adhesion. Colonization of cells to the biomaterial surface is a prerequisite for tissue integration. Successful cell colonization could be achieved by improved formation of focal adhesions, providing mechanical forces to the material surface (Grashoff et al., 2010). Vinculin, a highly conserved actin-binding protein, is frequently used as a marker for focal adhesion (Ohmori et al., 2010). Shown in **Figure 3B** is the CLSM micrograph exhibiting focal adhesion formation on different surfaces with all co-cultured systems. The vinculin protein was immunostained as the green background inside cells. In the PLGA/nHA group, we could observe relatively more focal adhesions formation in the presence of nHA compared to the PLGA group, which indicated that the nHA could improve cell colonization on the surface. Moreover, the cell adhesion on the PLGA/nHA/HACC (cell-first) group displayed confluent morphology with extensive actin filaments (red color) and the most abundant focal adhesions formation compared to all other groups due to the collaborative factors including preferential cell adhesion and the antibacterial activity of the bifunctional surface.

### Viability of Osteoblasts and *S. aureus* in Co-cultured Systems

**FIGURE 4:**
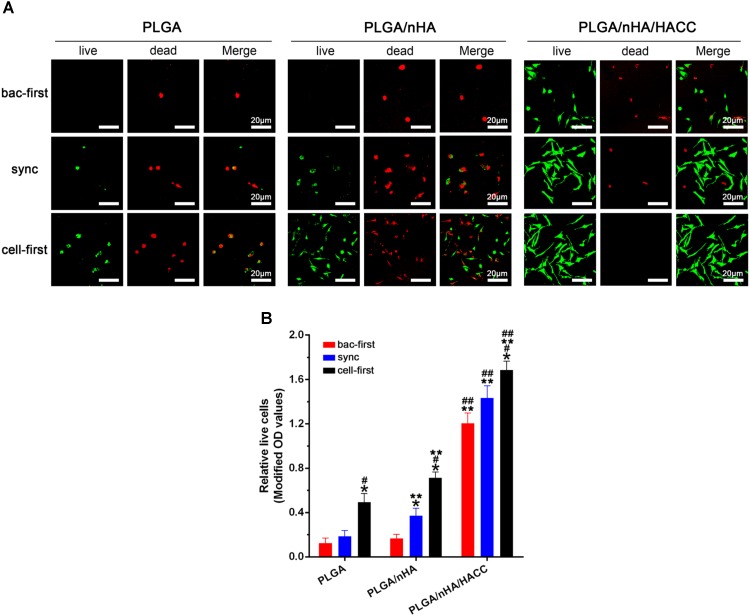
Qualitative and quantitative analyses of MC3T3-E1 cells on different materials surfaces in different co-cultured systems. **(A)** Qualitative analysis of cell viability on surfaces by the Live/Dead staining assay. Representative images of live cells (green fluorescence) and dead cells (red fluorescence). **(B)** Quantitative analysis of cell viability on surfaces by CCK-8 assay. ^∗^*p* < 0.05 compared with bac-first group on the same surface. ^#^*p* < 0.05 compared with sync group on the same surface. ^∗∗^*p* < 0.05 compared with PLGA group in the same co-cultured system. ^##^*p* < 0.05 compared with PLGA/nHA group in the same co-cultured system.

In the race of competitive colonization, the presence of *S. aureus* can diminish osteoblast viability by releasing cytotoxic products around cells (Perez-Tanoira et al., 2017). **Figure 4A** showed the cell viability on different surfaces with all the co-cultured systems determined by the Live/dead cell staining assay. The results showed that the cells were most viable in the PLGA/nHA/HACC (cell-first) group. The quantitative analysis by the CCK-8 assay also showed that cells were more prone to colonizing the surface in all the PLGA/nHA/HACC groups (*p* < 0.05, **Figure 4B**). Interestingly, **Figure 4B** showed that there were more live cells on the PLGA/nHA (sync, cell-first) than on the PLGA (sync, cell-first) surface, which suggests that preferential and improved cell adhesion could partially protect cells from bacterial toxicity on the surface. The colonized cells were relatively more stable, thus exhibiting a stronger immunity to external harmful factors in the co-cultured system. This could potentially improve tissue integration.

**FIGURE 5:**
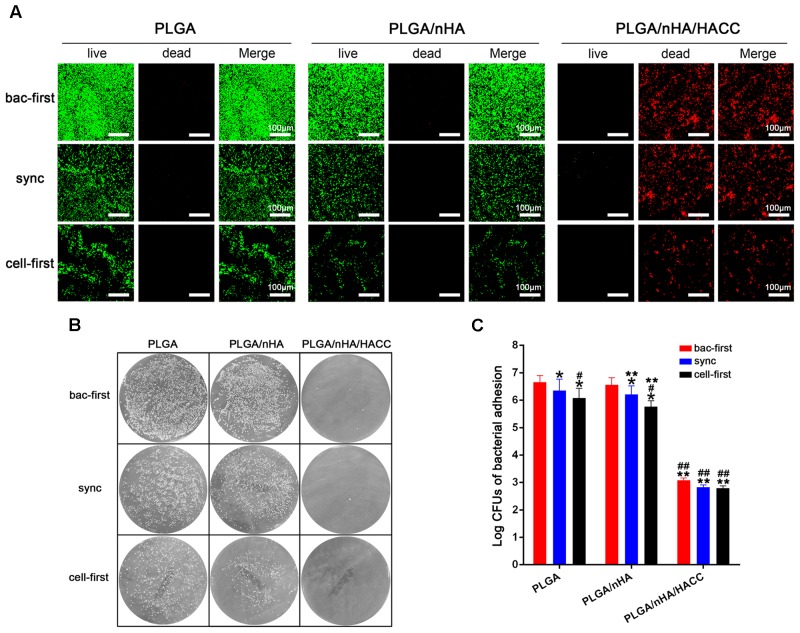
Qualitative and quantitative analyses of *S. aureus* on different materials surfaces in the different co-cultured systems. **(A)** Qualitative analysis of bacterial viability on surfaces by the Live/Dead BacLight viability kits. Representative images of live bacteria (green fluorescence) and dead bacteria (red fluorescence). **(B,C)** Quantitative analysis of bacterial viability on surfaces by the spread plate method. ^∗^*p* < 0.05 compared with bac-first group on the same surface. ^#^*p* < 0.05 compared with sync group on the same surface. ^∗∗^*p* < 0.05 compared with PLGA group in the same co-cultured system. ^##^*p* < 0.05 compared with PLGA/nHA group in the same co-cultured system.

The bacterial viability on different surfaces with all the co-cultured systems was tested using CLSM. As shown in **Figure 5A**, relatively less green fluorescence (green represents live bacteria) was observed on the PLGA (cell-first) and PLGA/nHA (cell-first) groups compared to the PLGA (bac-first, sync) and PLGA/nHA (bac-first, sync) groups, which suggests a low level of adherent bacteria on the surfaces after preferential cell colonization. We observed an intense red fluorescence (red represents dead bacteria) rather than green fluorescence for all PLGA/nHA/HACC groups due to the antibacterial activity of the bifunctional surface. The live bacteria were also quantified by the spread plate method, as shown in **Figures 5B,C**. The live bacteria in the PLGA (cell-first) and PLGA/nHA (cell-first) groups were significantly less than those in the PLGA (bac-first, sync) and PLGA/nHA (bac-first, sync) groups (*p* < 0.05). Moreover, the viable bacteria in PLGA/nHA/HACC with all three co-cultured systems was much lower than the other groups (*p* < 0.05). The stable bacterial colonization to biomaterial surface is considered to be the initial step toward the development of implant-related infection (Doll et al., 2017). The success of osteoblast colonization toward implant surfaces is thus of major importance for the deposition of bone matrix (Foss et al., 2015). The bifunctional surface developed here demonstrated the ability to facilitate osteoblast colonization in the co-existence of pathogens. This could be due to the synergistic effect of preferential cell adhesion and the antibacterial activity of the bifunctional surface.

### Analysis of Cytotoxicity Induced by Bacteria

**FIGURE 6:**
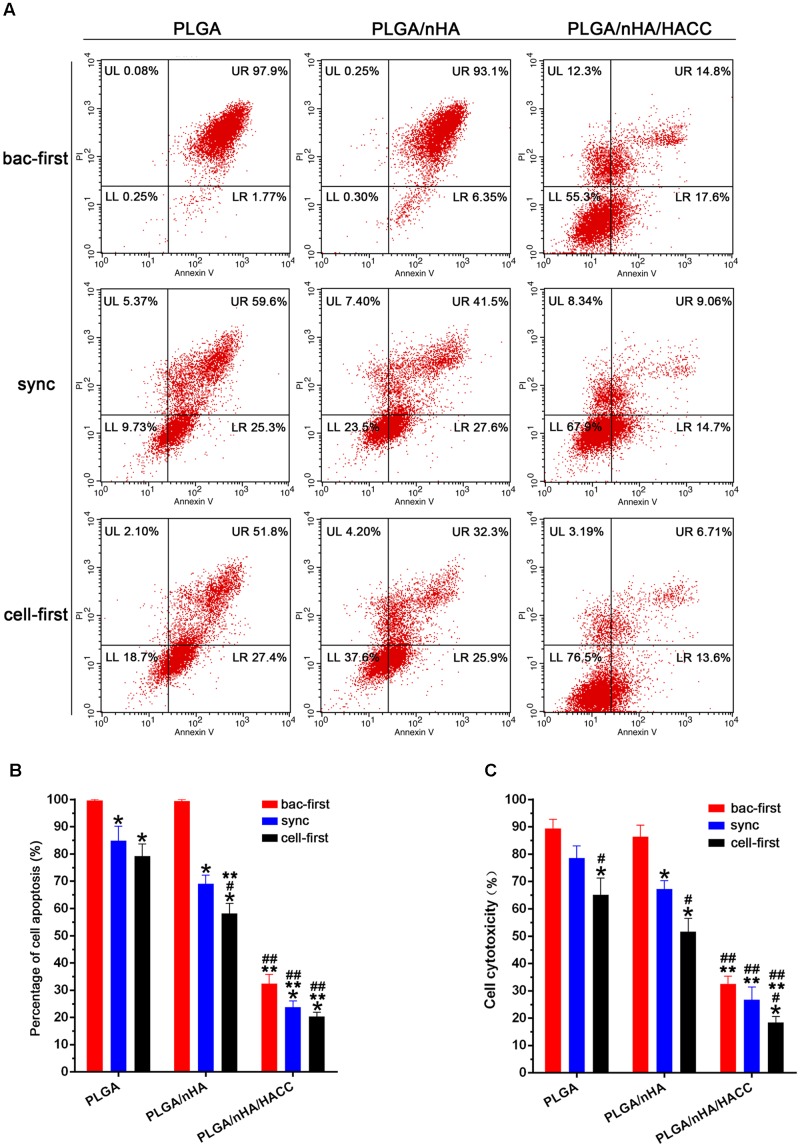
Analysis of cytotoxicity on different materials surfaces with different co-cultured systems. **(A,B)** Cell apoptosis assay with flow cytometry. **(C)** Cytotoxicity assessed by the release of LDH into the supernatant. ^∗^*p* < 0.05 compared with bac-first group on the same surface. ^#^*p* < 0.05 compared with sync group on the same surface. ^∗∗^*p* < 0.05 compared with PLGA group in the same co-cultured system. ^##^*p* < 0.05 compared with PLGA/nHA group in the same co-cultured system.

In the race of competitive colonization, the presence of bacteria can lead to a decreased cell viability (Perez-Tanoira et al., 2017). Bacteria are aggressive pathogens, which actively release cytotoxic products into the surrounding environment inducing apoptosis in osteoblasts. With our system the presence of bacteria could cause cell death, and this effect is believed to be impacted by both preferential adhesion and the antibacterial activity of the bifunctional surface (Flores et al., 2013). Preferential adhesion and rapid proliferation of bacteria may change the cellular microenvironment, making it unfavorable to sustain viability of cells. To understand the role of the cytotoxic effect of bacteria in the course of competitive colonization, we examined the rate of apoptosis of MC3T3-E1 cells by flow cytometry in all groups. The results shown in **Figures 6A,B** suggested that almost all cells were apoptotic in the PLGA (bac-first) and PLGA/nHA (bac-first) groups due to the toxic microenvironment produced by preferential bacterial adhesion. However, compared to the PLGA (bac-first) and PLGA/nHA (bac-first) groups, low apoptotic rates of cells were observed in PLGA (sync, cell-first) and PLGA/nHA (sync, cell-first) groups (**Figure 6B**) (*p* < 0.05). We deemed that the preferential cell colonization could partially protect cells from apoptosis in the co-cultured system. Importantly, the lowest cell apoptotic rate was found in the PLGA/nHA/HACC (cell-first) group compared to all other groups (**Figure 6B**, *p* < 0.05), which suggests that the synergistic effect of preferential cell adhesion bifunctional surface could effectively promote cell survival and suppress bacterial cytotoxicity to cells. Furthermore, we also assessed the release of LDH into the supernatant to evaluate the cytotoxicity in the co-cultured system (**Figure 6C**). The results were consistent with those of flow cytometry for all groups.

### Bacterial Biofilm Formation in Co-cultured Systems

**FIGURE 7:**
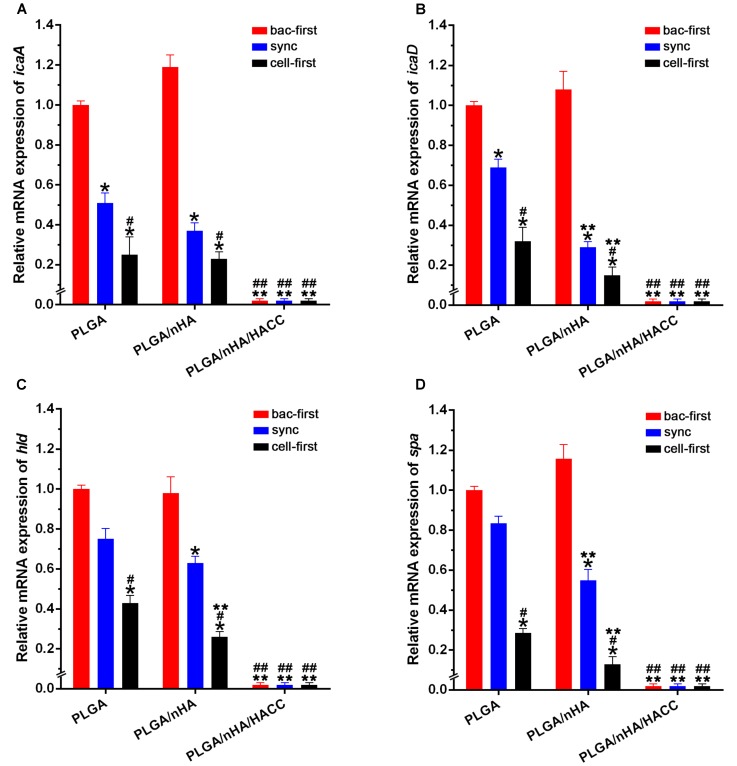
Real-time PCR analysis of bacterial biofilm-related gene transcription. **(A–D)** The expression levels of *icaA*, *icaD*, *hld* and *spa* normalized to the *16S rRNA* gene in biofilm formation by *S. aureus* on different materials surfaces in different co-cultured systems. The mRNA levels shown are the relative mRNA level compared to the mRNA level in the PLGA (bac-first) group. ^∗^*p* < 0.05 compared with bac-first group on the same surface. ^#^*p* < 0.05 compared with sync group on the same surface. ^∗∗^*p* < 0.05 compared with PLGA group in the same co-cultured system. ^##^*p* < 0.05 compared with PLGA/nHA group in the same co-cultured system.

Biofilm formation is thought to be a two-step process (Moormeier and Bayles, 2017; Xiang et al., 2017). First, the bacteria adhere to the surface, and then they form a complex biofilm architecture. The second step is referred to as the accumulative phase, which depends on the synthesis of the polysaccharide intercellular adhesion (PIA) encoded by the biofilm-related genes (Vu et al., 2009; Rohde et al., 2010). The *staphylococcus aureus* used in this study contains encoding genes of *icaA*, *icaD*, *hld* and *spa*, which are significant in the biofilm formation and pathogenesis of biomaterials-related infections (Tan et al., 2015). We assessed biofilm-related gene transcription levels as an index of biofilm formation on different biomaterials surface with the co-cultured systems by real-time PCR. The expression level of biofilm-related genes in all groups is shown in **Figure 7**. The biofilm-related gene expression level of *icaA*, *icaD*, *hld* and *spa* decreased dramatically in the PLGA (cell-first) and PLGA/nHA (cell-first) groups compared to the PLGA (bac-first, sync) and PLGA/nHA (bac-first, sync) groups (**Figure 7**, *P* < 0.05), which explains that preferential cell colonization could protect the biomaterial surface from bacterial adhesion, rapid bacterial growth and biofilm formation. Meanwhile, the lowest gene expression levels were observed for all PLGA/nHA/HACC groups compared to PLGA and PLGA/nHA groups (**Figure 7**, *P* < 0.05), which suggests that the antibacterial activity of the bifunctional surface could effectively prevent bacterial colonization, thus inhibiting biofilm formation. These results suggest that the synergistic action of preferential cell adhesion and the antibacterial activity of bifunctional surface can significantly prevent biofilm formation on the surface, thereby reducing bacterial viability.

## Conclusion and Perspectives

This study describes the development of different co-cultured systems in order to examine the ‘race for the surface’ theory. Such co-cultured systems offer a more comprehensive understanding on the competitive colonization between host cells and pathogens on a biomaterial surface. The system can be used as a guide to investigate implant-related infections and to test the real-world applicability of biomaterials. Meanwhile, the bifunctional PLGA/nHA/HACC composite used here showed resistance against bacterial adhesion and favored cell adhesion, making it a useful tool for the prevention of implant contamination by pathogens. We also demonstrated that the synergistic effect of preferential cell adhesion and the antibacterial activity of the bifunctional composite could favor cell survival as well as suppress bacterial cytotoxicity and biofilm formation on the biomaterial surface, which may significantly reduce bacterial adhesion. This study is useful for screening biomaterials under realistic conditions where both host cells and bacteria co-exist.

## Author Contributions

LC, YY, and TT conceived and designed the research. LC, YY, SY, QF, ZY, and X-LH conducted all the experiments and carried out data analyses. LC wrote the manuscript. X-PH, TDJ, and TT provided facilities, critically evaluated all the experiments and revised the manuscript. All the authors read and approved the final manuscript.

## Conflict of Interest Statement

The authors declare that the research was conducted in the absence of any commercial or financial relationships that could be construed as a potential conflict of interest.
